# Comparison of Percutaneous Achilles Tendon Lengthening and Open Z-Plasty in Children with Idiopathic Achilles Tendon Contracture

**DOI:** 10.3390/jcm15166208

**Published:** 2026-08-11

**Authors:** Piotr Morasiewicz, Anna Robinson, Łukasz Tomczyk, Krystian Kazubski, Marta Laszkowska Morasiewicz, Paweł Leyko

**Affiliations:** 1 Department of Orthopaedic and Trauma Surgery, Institute of Medical Sciences, University of Opole, al. Witosa 26, 45-401 Opole, Poland; 2 Institute of Medical Sciences, University of Opole, ul. Oleska 48, 45-052 Opole, Poland; 3 Department of Food Safety and Quality Management, Poznan University of Life Sciences, Wojska Polskiego 31, 60-624 Poznan, Poland; 4 Faculty of Medicine, Institute of Medical Sciences, University of Opole, ul. Oleska 48, 45-052 Opole, Poland

**Keywords:** Achilles tendon, Achilles lengthening, percutaneous lengthening, Z-plasty, ankle dorsiflexion

## Abstract

**Background:** Achilles tendon contracture is a prevalent issue in pediatric populations, often leading to complications such as toe walking and limited ankle dorsiflexion. This study aims to compare the clinical outcomes of two surgical techniques for Achilles tendon lengthening—conventional percutaneous lengthening and Z-plasty—in children with idiopathic Achilles tendon contracture, while assessing the adequacy of a six-week postoperative immobilization period. **Methods:** A retrospective analysis was conducted on 60 pediatric patients (ages 5–17) who underwent Achilles tendon lengthening at our institution between 2021 and 2025. Patients were divided into two groups: 25 receiving percutaneous lengthening and 35 undergoing Z-plasty. Inclusion criteria encompassed idiopathic contracture with a follow-up of over 12 months. Outcomes measured included ankle dorsiflexion, time to resume normal activities, length of hospital stay, duration of cast immobilization, complications, the likelihood of choosing the same treatment method again, taking analgesics, AOFAS score, and patient/guardian satisfaction. **Results:** Both surgical methods resulted in significant improvements in ankle dorsiflexion and functional outcomes. The percutaneous group demonstrated a mean increase of 30° in dorsiflexion, while the Z-plasty group showed a mean increase of 32.5°. The time from surgery to resuming normal activities in the Z-plasty group ranged from 6 to 11 weeks, which was significantly higher than the range of 6 to 7 weeks in the percutaneous lengthening group, *p* = 0.024. Guardian and patient satisfaction was high in both groups, with 94.3% of patients from the Z-plasty group and 100% of patients from the percutaneous group expressing willingness to choose the same treatment again. Four patients (11.4%) from the Z-plasty group and one patient (4%) from the percutaneous lengthening group developed complications. **Conclusions:** The period of cast immobilization of six weeks helped achieve good outcomes and caused few complications in children from both study groups. The conventional percutaneous approach to Achilles tendon lengthening in children helps achieve substantial correction of ankle dorsiflexion with a low risk of complications. Due to the risk of selection bias, the results should be interpreted with caution.

## 1. Introduction

Achilles tendon contracture is a common problem, particularly in children [1,2,3,4,5]. Achilles tendon lengthening surgery is one of the most common surgical procedures in children [3,5,6]. Idiopathic toe walking occurs in approximately 1–5% of children [1]. The etiology of Achilles tendon contracture may vary, including idiopathic, neurogenic, posttraumatic, postinflammatory, or metabolic causes [7,8,9,10,11,12]. Achilles tendon contracture manifests with toe walking, absent ankle dorsiflexion, limping, equinus foot deformity, pain, balance problems, corns, weakened muscles, and psychological problems [1,2,3,5,8,9,11]. Achilles tendon contracture in children can disrupt the development of normal foot anatomy. During the growth period, such a contracture can lead to developing foot deformities, most commonly including clubfoot (equinovarus), flat-valgus foot, and serpentine foot. Persistent shortening of the gastrocnemius–soleus–Achilles tendon complex alters normal ankle biomechanics, limits dorsiflexion, disrupts gait, and increases plantar forefoot loading, ultimately impairing mobility, balance, and overall quality of life [1,2,3,5,8,9,11]. If left untreated, equinus deformity may lead to compensatory changes throughout the kinetic chain, including knee hyperextension, excessive hip flexion, pelvic malalignment, and secondary foot deformities [3,5,7,9,11]. Pathological Achilles tendon remodeling occurs when the balance between matrix synthesis and degradation is disrupted [2,4,11,12]. Chronic overload, prolonged immobilization, altered muscle tension, or congenital neuromuscular abnormalities may promote excessive collagen cross-linking, extracellular matrix disorganization, fibrosis, and reduced tendon elasticity. These structural changes progressively decrease tendon extensibility, contributing to fixed contracture and impaired ankle joint mobility [2,3,4,11,12]. Achilles tendon contracture requires treatment due to its detrimental impact on the musculoskeletal system. The treatment for Achilles tendon contracture includes, initially, conservative treatment (exercises, rehabilitation, swimming pool activities) for a period of 3–6 months, and—if there is no improvement (normalized ankle dorsiflexion)—surgical treatment is recommended [1,2,3,11]. An equinus foot deformity can result from a contracture of the Achilles tendon and/or the gastrocnemius–soleus complex. Gastrocnemius recession is often recommended for patients with limited ankle dorsiflexion caused by tight calf muscles. Gastrocnemius release can be performed using various techniques (for example, the Vulpius, Strayer, or Baumann procedures) [1,2,7,9,10,11,12].

The most commonly used methods of Achilles tendon lengthening include Z-plasty and percutaneous techniques [1,2,7,9,10,11,12]. There is no consensus regarding the preferred method of Achilles tendon lengthening [5,6,8,13,14,15,16]. Some authors report the superiority of percutaneous techniques [1,2,3,11,12,15,16] due to their minimally invasive nature and low complication rates. Conversely, some studies show that percutaneous lengthening improves dorsiflexion only to a limited extent and may be associated with the risk of nerve or vascular damage, Achilles tendon rupture, calcaneal gait, and deformity recurrence [2,3,12,15,16]. Achilles tendon Z-plasty allows for a considerable increase in ankle dorsiflexion; however, it is associated with a risk of complications (mainly soft tissue adhesions, pain, deformity recurrence, Achilles tendon rupture) and leaves a large postoperative scar [2,3,5,6,11,12,16]. In comparison with percutaneous techniques, Z-plasty helps more precisely determine the extent of the necessary Achilles tendon lengthening [6].

The issue of comparing the effectiveness of various Achilles tendon lengthening techniques has not been fully investigated. There have been only a handful of studies that compare various methods of Achilles tendon lengthening [2,5,12]. All available reports compare the various methods of lengthening the Achilles tendon in mixed-age populations (children and adults) [2,5,12]. There are no studies that compare various methods of Achilles tendon lengthening in children specifically. The period of postoperative immobilization may affect the outcomes. Some authors suggest that longer postoperative immobilization (in a cast or brace) may limit joint mobility, cause swelling, pain, soft-tissue adhesions, difficulty walking, or muscle atrophy [1,3]. Some authors recommend postoperative immobilization for a long time (8–12 weeks) [2,11,12]. Currently, a large proportion of authors recommend modified percutaneous Achilles tendon lengthening techniques to minimize the risk of complications and lengthened tendon rupture [2,11,12]. Modified percutaneous Achilles tendon lengthening techniques have been reported to be more complicated, and thus may be associated with the risk of vascular or nerve damage, take longer to perform, and require a more experienced operator [2,11,12].

In the available literature there are no papers comparing different methods of Achilles tendon lengthening in children with idiopathic Achilles tendon contracture. Available papers on this topic resulted from studies assessing Achilles tendon lengthening due to idiopathic deformity by means of a single lengthening method [1,6,9]. The etiology of the contracture may affect treatment outcomes [9,12,15].

Considering the above, we posited the following hypotheses: (1) various surgical techniques of Achilles tendon lengthening in children yield comparable outcomes; (2) six weeks of immobilization after Achilles tendon lengthening in children is sufficient. The purpose of our study was to compare the effectiveness of Achilles tendon lengthening in children with idiopathic Achilles tendon contracture by means of various techniques (conventional percutaneous lengthening and “Z-plasty”) followed by a six-week period of immobilization.

## 2. Materials and Methods

The purpose of this retrospective study was to assess patients following various Achilles tendon lengthening procedures conducted at our department in 2021–2025. Study inclusion criteria were lack of improvement in ankle dorsiflexion after 6 months of conservative treatment, a negative Silfverskiöld test, ankle dorsiflexion of less than 10° [1,2,8,11], Achilles tendon percutaneous lengthening or Z-plasty, idiopathic Achilles tendon contracture, age of 5–17 years, a follow-up of over 12 months, complete medical and radiological records, informed assent, the absence of other lower limb abnormalities, and complete follow-up medical records. Study exclusion criteria were non-idiopathic (neurogenic, traumatic, postinflammatory, or metabolic) Achilles tendon contracture, a follow-up period of less than 12 months, absence of informed assent, incomplete medical and radiographic records, other lower-limb surgeries, other lower-limb abnormalities, and incomplete records from the long-term follow-up visit. The Achilles tendon contracture was considered idiopathic only when no identifiable underlying cause was found. Patients with a history or clinical signs suggestive of neurological disorders, neuromuscular diseases, genetic syndromes, previous lower-limb trauma, surgery, or other conditions known to cause secondary Achilles tendon contracture were excluded. When indicated by the medical history or clinical examination, patients underwent specialist neurological evaluation. Therefore, only patients without evidence of neurological or syndromic disorders were included in the study. The study had been approved by the local ethics committee. The study was conducted in accordance with the Declaration of Helsinki. All evaluated patients and their legal guardians were informed of the voluntary nature of the study and of the possibility to withdraw from the study at any time. All patients and their legal guardians provided their assent/consent to participate in the study.

In the years 2021–2025, a total of 89 Achilles tendon lengthening procedures were conducted at our center. Application of the inclusion and exclusion criteria yielded a total of 60 patients (65 Achilles tendons were lengthened, with 5 patients undergoing bilateral tendon lengthening). There were 25 patients (5 females, 20 males) in the percutaneous lengthening group, and 35 patients (6 females, 29 males) in the Z-plasty group. A total of 29 patients were excluded from the study—two children had incomplete medical and radiological records, 15 had non-idiopathic Achilles tendon contracture, 10 had a follow-up period of less than 12 months, and two patients were lost to follow-up.

All patients had undergone the Silfverskiöld test, and their ankle dorsiflexion was less than 10° [1,2,8,11]. All patients underwent an X-ray of the foot and ankle joint prior to treatment to rule out synostoses and other bone conditions [2,6,9,11]. None of the patients had achieved a normal range of ankle dorsiflexion following conservative treatment (exercise, rehabilitation, swimming pool) for 6 months.

All patients were operated on by two experienced orthopedic surgeons. The surgical technique was chosen based on the operator’s preference. One of the orthopedic surgeons preferred percutaneous tendon lengthening, which he performed on all his patients, whereas the other orthopedic surgeon preferred Z-plasty, which he performed on all his patients. All surgeries were performed in a supine position. Percutaneous tendon lengthening was done with the standard Hoke’s method [2,3,16]. Three short (2–4 mm) skin incisions approximately 2 cm apart were performed along the tendon with scalpel blade No. 15. The most distal incision was performed approximately 1 cm proximal to the calcaneal tuberosity. A cut of the medial half of the Achilles tendon diameter was made through the proximal and distal incisions after rotating the blade by 90 degrees, whereas the middle skin incision was used to cut the lateral half of the Achilles tendon diameter after rotating the blade by 90 degrees. Subsequently, forcible ankle dorsiflexion was manually applied to the foot. Once the Achilles tendon was lengthened (an audible “pop” of the tendon and improved ankle dorsiflexion), the skin incisions were closed with sutures, and the limb was immobilized in a short leg cast, with the foot and ankle joint at a neutral angle.

Z-plasty was performed with the conventional technique [2,6,16], with the patient in a supine position, with a tourniquet placed on the treated limb. A skin incision was made; it extended from 1 cm proximal to the calcaneal tuberosity approximately to the myotendinous junction [6]. The Z-plasty technique of Achilles tendon lengthening was performed. Once a more natural foot position was achieved and ankle dorsiflexion was increased, the Achilles tendon was sutured at a neutral position of the ankle joint and foot [2]. The ankle was immobilized in this position in a short leg cast.

Routinely, all patients from both groups received a short leg cast for 6 weeks and were instructed to walk using two elbow crutches, with partial weight-bearing on the treated foot. Once the cast was removed, the patients were allowed to walk without crutches, with full weight-bearing on the treated foot. As is standard, the patients were then allowed to resume physical activities, sports, and participation in physical education classes. Foot dorsiflexion and plantar flexion exercises up to the pain threshold were recommended. Typically, postoperative rehabilitation was not recommended [1,9,13]. This approach is based on clinical experience and the specific characteristics of the pediatric population, in whom a spontaneous, rapid recovery of range of motion and limb function during daily activities is typically observed following the healing of the Achilles tendon [1,9,13]. Follow-up visits took place initially every 6 weeks and subsequently every 3 months.

At least 12 months after treatment completion, we assessed the available medical and radiological records, follow-up physical examination findings, functional scale scores, and the following parameters: the time from surgery to resuming normal activity, the length of hospital stay, duration of cast immobilization, the likelihood of choosing the same treatment method again, guardian’s satisfaction with treatment, patient’s satisfaction with treatment, complications, equinus deformity recurrence, Achilles tendon rupture, calcaneal deformity, normal gait, taking analgesics, American Orthopaedic Foot and Ankle Society (AOFAS) scale score, type of footwear, and foot mobility.

The time from surgery to resuming normal activities was defined as the time from surgery to returning to school or kindergarten and walking without crutches; this was expressed in weeks. The length of hospital stay was counted from the admission date to the discharge date; this was expressed in days [2]. The duration of cast immobilization was counted from cast placement to removal; this was expressed in weeks. The likelihood of a patient’s willingness to choose the same method of treatment again was assessed [17]. The level of satisfaction with treatment was assessed separately for the parent/guardian and the patient, based on a subjective rating of *unsatisfied*, *satisfied*, or *very satisfied* [17].

Complications were assessed based on history taking (from both the patient and the guardian), analysis of medical and radiological records, and physical examination. The following complications were considered: deformity/equinus recurrence, pain, Achilles tendon rupture, swelling, infection, delayed wound healing, soft-tissue necrosis, adhesion, scarring, calcaneal deformity, nerve and vascular damage, revision surgery, and joint instability.

Based on history-taking conducted with the patient and the parent/guardian, medical records assessment, and long-term follow-up physical examination, the possibility of normal gait was assessed prior to surgery and at long-term follow-up. Abnormal gait was defined as toe walking, limping, or calcaneal gait [1].

The use of painkillers was assessed based on the available medical records and history [17]. Specifically, we assessed the use of paracetamol, ibuprofen, metamizole, nonsteroidal anti-inflammatory drugs, tramadol, and opioids prior to surgery and at long-term follow-up. AOFAS scale scores were evaluated prior to surgery and at long-term follow-up. The AOFAS scale helps assess pain and the function and position of the foot and ankle joint. Based on history taking, review of medical records, and follow-up physical examination, we assessed whether the patient used normal footwear prior to surgery and at long-term follow-up [1]. Review of medical records and follow-up examination findings showed the extent of foot mobility prior to surgery and at long-term follow-up. The physical examination was conducted with a goniometer in a supine position, with the lower limb extended at the knee, and maximum passive plantar flexion and dorsiflexion of the foot were measured [1,15]. The obtained results were compared between patients from the Achilles tendon “Z-plasty” group and the percutaneous Achilles tendon lengthening group.

### Statistical Analysis

Statistical analysis was performed using Statistica 14.1 software (TIBCO Software Inc., Palo Alto, CA, USA). The distribution of continuous variables was assessed using the Shapiro–Wilk test. Since most variables did not meet the assumption of normal distribution, non-parametric methods were applied. Continuous variables were presented as medians and interquartile ranges (Q1–Q3), whereas categorical variables were expressed as numbers and percentages. Comparisons between the Z-plasty group and the percutaneous group were performed using the Mann–Whitney U test for continuous variables and Pearson’s chi-square test (or Fisher’s exact test when appropriate) for categorical variables. Changes between preoperative assessment and the last follow-up within the same group were analyzed using the Wilcoxon signed-rank test for continuous variables and the exact McNemar test for paired categorical variables. Effect size for the Wilcoxon signed-rank test was calculated as r (r = |Z|/√N). For paired binary outcomes analyzed with the McNemar test, effect size was expressed as Cohen’s g, calculated as g = |b − c|/N, where b and c represent the numbers of discordant pairs. Additionally, an approximate conversion to Cohen’s d was reported for non-parametric comparisons. A *p*-value < 0.05 was considered statistically significant.

## 3. Results

The median follow-up in the Z-plasty group was 26 months (12–57 months), whereas the median follow-up in the percutaneous lengthening group was 33 months (12–55 months); the difference between the groups in terms of the duration of follow-up was not statistically significant; see Table 1. The median age at the time of surgery was 11 years (5–17 years) in the Z-plasty group and 10 years (5–14 years) in the percutaneous lengthening group; the difference between the groups was not statistically significant; see Table 1.

**Figure 1:**
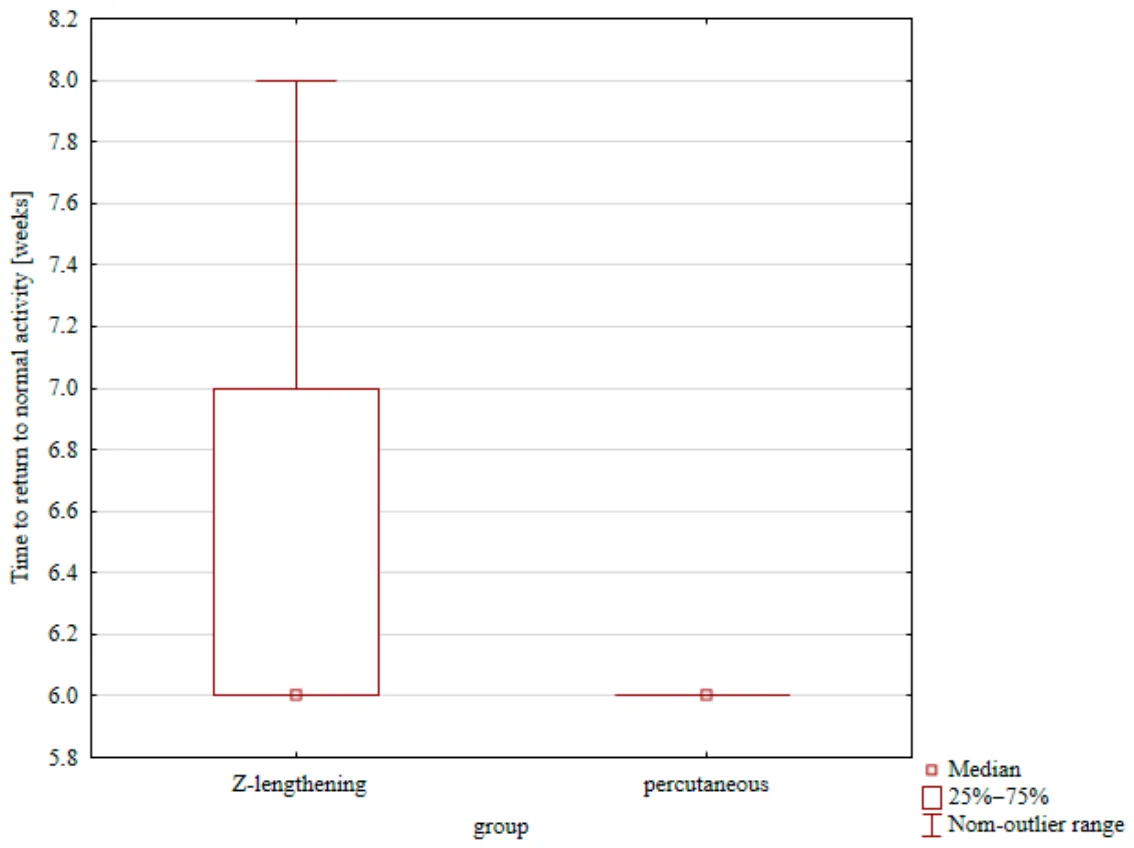
Time until resuming normal activities in the two groups.

The two groups did not differ significantly in terms of the length of hospital stay (the median length of stay in both groups was 3 days); see Table 1. The median duration of cast immobilization in the Z-plasty group was six weeks, and in the percutaneous lengthening group it was six weeks; there was no significant difference between the two groups; see Table 1. The time from surgery to resuming normal activities in the Z-plasty group ranged from 6 to 11 weeks, which was significantly higher than the range of 6 to 7 weeks in the percutaneous lengthening group (*p* = 0.024; Table 1, Figure 1). The median of both groups for these periods was 6 weeks.

In our study, 94.3% of patients from the Z-plasty group and 100% of patients from the percutaneous group declared a willingness to choose the same treatment method again; the difference was not significant; see Table 1. Assessment of guardian satisfaction with treatment in the Z-plasty group revealed 77.1%, 20%, and 2.9% of guardians to be very satisfied, satisfied, and unsatisfied with treatment, respectively. In the percutaneous lengthening group, 92% and 8% of guardians were very satisfied and satisfied with treatment, respectively. There were no significant differences between the groups in terms of guardian satisfaction with treatment; see Table 1. In terms of patient satisfaction in the Z-plasty group, 68.6%, 28.6%, and 2.8% of patients were very satisfied, satisfied, and unsatisfied with treatment, respectively. In the percutaneous lengthening group, 88% and 12% of patients were very satisfied or satisfied with treatment, respectively. The difference in the proportion of very satisfied and satisfied patients did not differ significantly between groups; see Table 1. All assessed patients from either group used normal footwear both prior to treatment and at follow-up.

Four patients (11.4%) from the Z-plasty group and one patient (4%) from the percutaneous lengthening group developed complications; the difference was not statistically significant; see Table 1. Two patients (5.7%) from the Z-plasty group developed equinus recurrence, which was treated conservatively with exercise and rehabilitation; two patients reported pain in the foot and ankle joint, which resolved after administration of analgesics, ice compresses, and rehabilitation. One patient from the percutaneous lengthening group reported foot and ankle joint pain, which resolved after administration of analgesics, ice packs, and rehabilitation. None of the evaluated patients developed Achilles tendon rupture, calcaneal deformity, swelling, infection, delayed wound healing, soft-tissue necrosis, adhesion, scarring, nerve and vascular damage, or joint instability.

Abnormal gait prior to surgery was noted in all patients from both evaluated groups. Post-treatment follow-up showed abnormal gait in two patients (5.7%) from the Z-plasty group and in none of the patients from the percutaneous lengthening group; this difference was not significant; see Table 1. The reduction in the proportion of patients with abnormal gait from before to after treatment was significant in both evaluated groups (*p* < 0.001; Table 2).

Taking painkillers before surgery was noted in 11.4% of patients from the Z-plasty group and in 4% of patients from the percutaneous lengthening group; this difference was not significant; see Table 1. At post-treatment follow-up, none of the patients from either group was taking painkillers; see Table 1. We observed no significant difference between the proportion of patients taking painkillers prior to treatment and at post-treatment follow-up in either the Z-plasty group or the percutaneous lengthening group; see Table 2.

**Figure 2:**
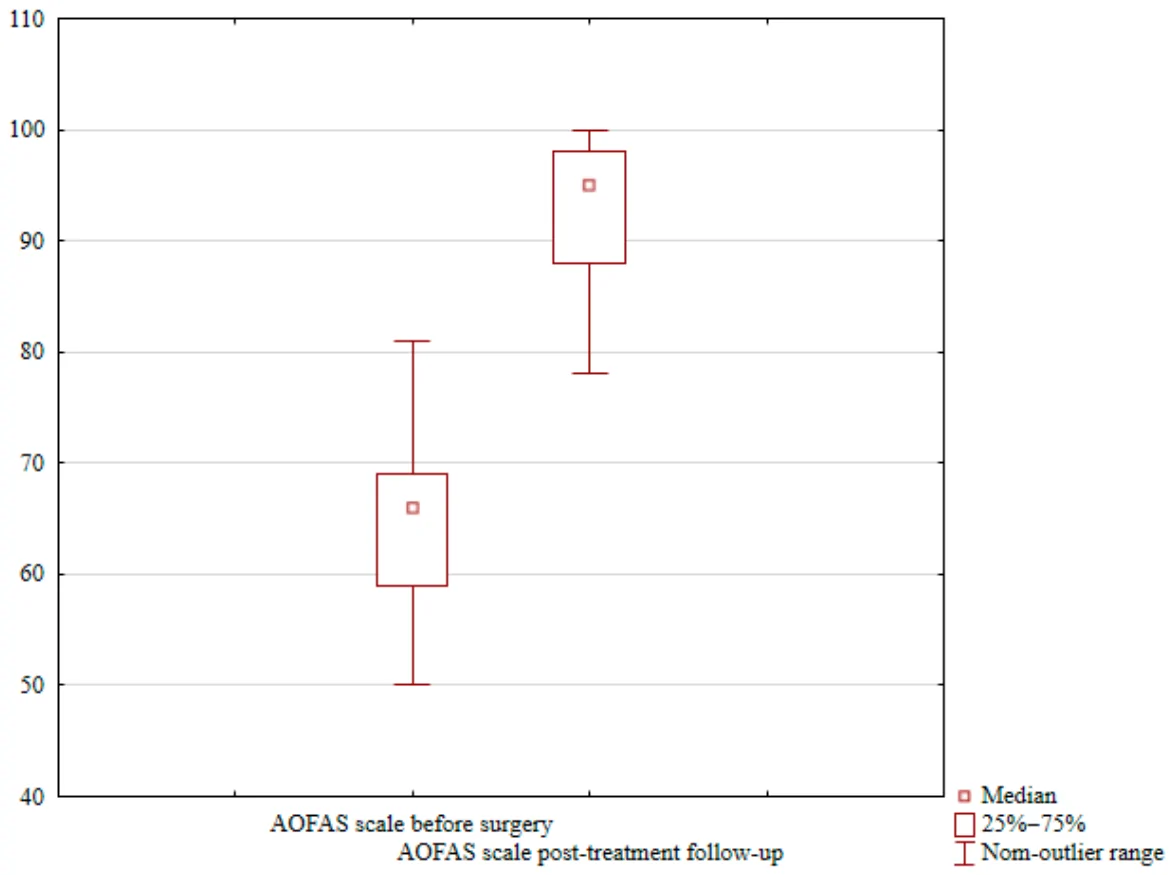
AOFAS scale scores before surgery and in post-treatment follow-up in the Z-plasty group. A significant improvement in postoperative AOFAS scale scores was also observed in the percutaneous lengthening group (*p* < 0.001; Table 2, Figure 3).

The median preoperative AOFAS scale scores were 66 and 65 points in the Z-plasty group and in the percutaneous lengthening group, respectively; the difference was not statistically significant; see Table 1. The median AOFAS scale scores at follow-up in both groups were 95 points; there was no significant difference; see Table 1. A comparison of AOFAS scale scores prior to and after treatment revealed a significant improvement in the Z-plasty group (*p* < 0.001; Table 2, Figure 2).

The median preoperative ankle dorsiflexion values in the Z-group and in the percutaneous group were −7.5° and 0°, respectively; this was a significant difference (*p* = 0.02; Table 1). At follow-up, the median ankle dorsiflexion was 25° in the Z-plasty group and 30° in the percutaneous lengthening group; this difference was statistically significant (*p* = 0.047; Table 1).

**Figure 4:**
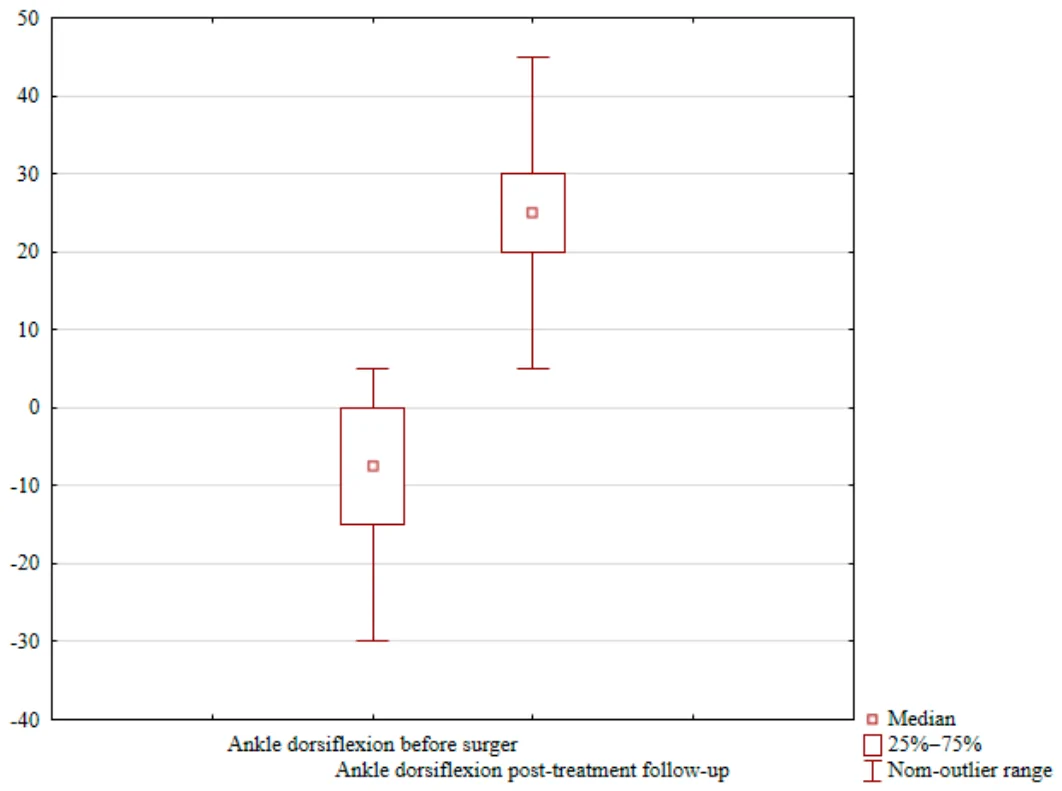
Ankle dorsiflexion before surgery and in post-treatment follow-up in the Z-plasty group. In patients who underwent percutaneous lengthening of the Achilles tendon there was also a significant improvement in ankle dorsiflexion at follow-up in comparison with preoperative values (*p* < 0.001; Table 2, Figure 5).

Ankle dorsiflexion assessment in the Z-plasty group revealed a significant improvement in the follow-up values in comparison with preoperative values (*p* < 0.001; Table 2, Figure 4).

**Figure 5:**
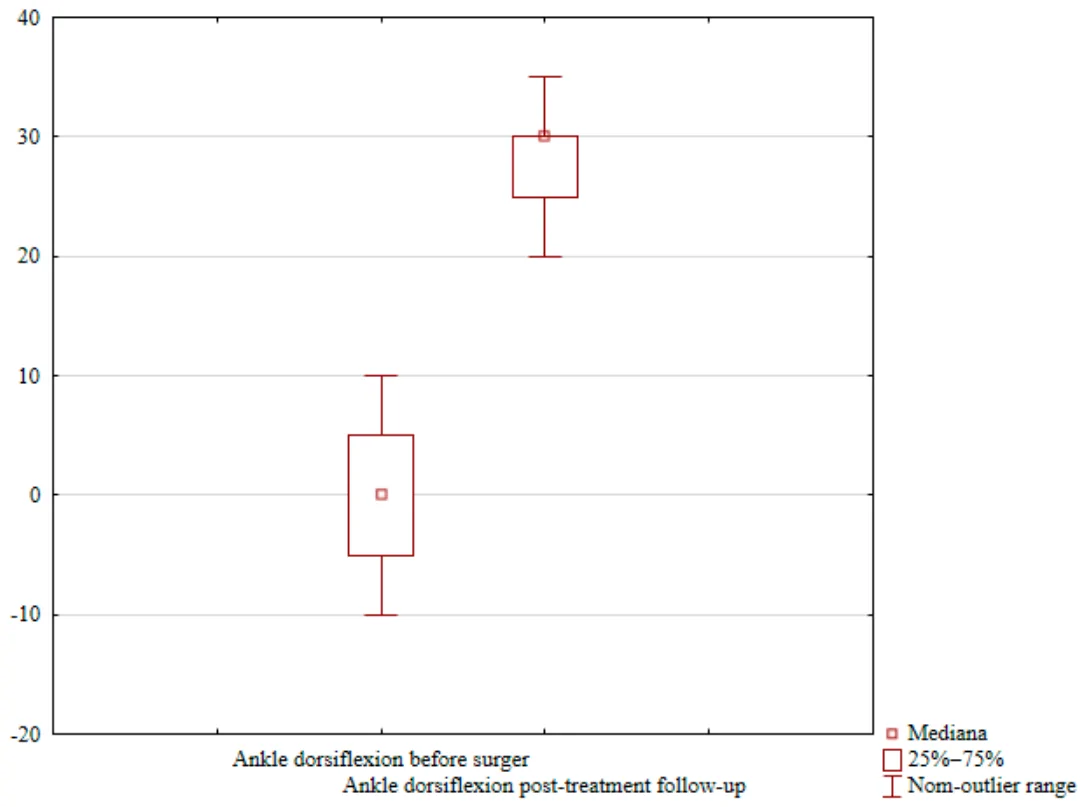
Ankle dorsiflexion before surgery and in post-treatment follow-up in the percutaneous lengthening group.

## 4. Discussion

Our study demonstrated that the conventional percutaneous technique is an effective treatment method yielding good outcomes and that a six-week period of immobilization in a cast following Achilles tendon lengthening in children is sufficient. A comparison between study groups showed a shorter time until resuming normal activities only in patients who underwent percutaneous Achilles tendon lengthening. Our study results support the research hypothesis.

Some authors suggest that the conventional percutaneous technique of Achilles tendon lengthening is imprecise, hardly effective, and is associated with a high risk of complications [2,11,12,13,15,16]. According to those authors, the conventional percutaneous technique allows for correcting ankle dorsiflexion only to a limited degree and is associated with the risk of Achilles tendon rupture, nerve or vascular damage, pain, or infection [2,11,12,13,15,16]. As a result, modified percutaneous techniques of Achilles tendon lengthening were introduced [2,11,12]. However, modified percutaneous lengthening techniques have drawbacks in the form of longer procedure duration, a higher level of technical difficulty in performing the procedure, a demand for a more experienced operator, and a greater risk of damage to blood vessels or nerves in comparison with the conventional percutaneous technique [2,11,12]. Postoperative ultrasonography has revealed Achilles tendon thinning, structural alterations, and hypoechoic areas after lengthening surgery (either with a percutaneous technique or with Z-plasty) [4]. As the Achilles tendon recovers following its lengthening, there may be proliferation of abnormal tenocytes, disruption of collagen fibers, and an increase in non-collagenous matrix, which may increase the risk of tendon rupture [4]. Our theoretical and practical experience shows that the conventional percutaneous technique for Achilles tendon lengthening still yields good outcomes and offers a sufficient correction of the range of foot motion in children; these conclusions have been substantiated by the results of this study. None of the patients who underwent a conventional percutaneous lengthening in our study developed Achilles tendon rupture, deformity recurrence, pain, or swelling, which indicates good treatment outcomes. Achilles tendon blood supply is the poorest at 2–6 cm proximal to its distal point of attachment [16]. Some authors suggest that the conventional percutaneous technique, with the incisions performed less than 5 cm apart, increases the risk of further limiting the blood supply and, thus, the risk of Achilles tendon rupture [2,11,12,16]. In the group that underwent conventional percutaneous Achilles tendon lengthening, there were no cases of impaired blood supply or Achilles tendon rupture. We believe that there is no reason to use a more complicated, modified percutaneous technique of Achilles tendon lengthening in children.

The main objective of Achilles tendon lengthening is to improve ankle dorsiflexion (i.e., achieving a minimum plantigrade foot position) and foot function [14]. Our study results showed that this main objective was achieved in all patients undergoing percutaneous lengthening and in 94.3% of patients undergoing Z-plasty.

On the one hand, a number of authors report that a short period of immobilization following Achilles tendon lengthening may result in complications; particularly, it may increase the risk of tendon rupture [2,11,12]. On the other hand, prolonged postoperative immobilization is detrimental, since it may result in muscle atrophy, soft tissue adhesions, pain, swelling, walking difficulties, reduced level of satisfaction with treatment, and limited joint mobility [1,3]. Some authors recommend long immobilization of up to 8–12 weeks following Achilles tendon lengthening (either with the percutaneous technique or Z-plasty) [2,11,12,13]. Others report that six weeks of immobilization after Achilles tendon lengthening is enough to achieve cell proliferation and tissue maturation sufficient for preserving the necessary strength of the Achilles tendon [16]. Our experience suggests that short leg cast immobilization for a period of six weeks following Achilles tendon lengthening (either with a percutaneous technique or with Z-plasty) was associated with satisfactory outcomes in this cohort. We believe that there is no reason to extend the total period of immobilization (with a cast or brace) over six weeks. The median cast immobilization in both groups in our study was six weeks. Our study results supported our hypothesis about the duration of immobilization. None of our patients suffered Achilles tendon rupture, deformity recurrence, pain, or swelling, which are potential complications associated with short-term immobilization. We believe that a six-week immobilization in a short leg cast allows for early rehabilitation and patient mobilization. This helps reduce complications, such as muscle atrophy and joint mobility limitations. A shorter period of cast immobilization of up to 4–5 weeks may be considered after percutaneous lengthening. Such a shorter immobilization period might improve patient and guardian satisfaction with treatment, and would be unlikely to increase the risk of complications [3].

Congenital lower-limb deformities, including clubfoot (congenital talipes equinovarus), congenital vertical talus, arthrogryposis multiplex congenita, and neuromuscular disorders such as cerebral palsy, are frequently associated with Achilles tendon shortening [2,11,12,13,16,18]. In these conditions, abnormal musculoskeletal development, muscle imbalance, spasticity, or connective tissue abnormalities interfere with normal tendon growth and remodeling, resulting in progressive equinus deformity [2,3,5,7,11,12,13,16,18]. From a biomechanical perspective, the Achilles tendon lengthening procedure increases ankle dorsiflexion by reducing posterior forces acting on the subtalar and tibiotalar joints [3,5,7,11,15].

There have been only a handful of studies in which the period of hospital stay following Achilles tendon lengthening was assessed. In the population evaluated by Lin et al., the mean duration of hospital stay was 3.08 days and 6.46 days in the percutaneous lengthening group and in the Z-plasty group, respectively [2]. In our study, the mean duration of hospital stay in both groups was 3 days, which was a better result for the Z-plasty group than those reported in the literature [2].

There are no studies in the available literature assessing the important parameter of the patient’s willingness to choose the same treatment method in patients who undergo Achilles tendon lengthening. Leyko et al. claimed that all pediatric patients who had undergone hemiepiphysiodesis declared a willingness to choose the same treatment method again [17]. In our study, 94.3% of Z-plasty patients and 100% of percutaneous lengthening patients declared a willingness to choose the same treatment method again, which indicates their satisfaction with treatment outcomes and is a good result.

There are no studies assessing the length of time from surgery to resuming normal activities in patients who had undergone Achilles tendon lengthening. In comparison with the Z-plasty group, the patients who underwent percutaneous lengthening in our study were characterized by a significantly shorter time until resuming their normal activities. This may be due to a larger surgical scar and a greater extent of the procedure or more severe postoperative pain.

There are few authors who have assessed the level of parental satisfaction with Achilles tendon lengthening in their children [1], despite this being one of the most important aspects of treatment outcome evaluation. Kha et al. assessed 19 patients who had undergone percutaneous Achilles tendon lengthening, and all parents were satisfied with the treatment [1]. Zhang et al. reported satisfaction with treatment in 93.9% of patients who had undergone Achilles tendon lengthening [11]. Our study showed that all patients who had undergone percutaneous lengthening as well as their guardians were satisfied or very satisfied with the treatment outcome. In the Z-plasty group, 97.1% of patients and guardians were satisfied or very satisfied with treatment, which indicates good subjective treatment outcomes. Such high levels of satisfaction among both patients and their guardians may be a result of the relatively short postoperative immobilization (6 weeks) and good aesthetic outcomes of treatment.

Kha et al. reported complications in 21% of patients with Achilles tendon contracture treated with the percutaneous lengthening technique [1]. The total complication rate was 8.3% in the percutaneous group and 35.4% in the Z-plasty group [2]. Hao et al. reported complications in 12.5% of patients who had undergone endoscopic lengthening of the Achilles tendon [10]. Zhang et al. observed complications in 12.1% of patients treated with percutaneous lengthening [11]. Berg et al. reported a 13% complication rate after percutaneous lengthening [13]. Hemo et al. reported a 13.3% complication rate [15]. According to Dietz et al., one of the most common complications following Achilles tendon lengthening is over-lengthening, manifesting with calcaneal gait [5]. There were no cases of calcaneal gait among patients from either of the groups evaluated in our study. A paper by Ozyalvec et al. contained a complicated and time-consuming mathematical formula for assessing the necessary extent of Achilles tendon lengthening [6]. Those authors claimed that such mathematical assessment is recommended to limit the risk of an insufficient or too extensive Achilles tendon lengthening [6]. We believe that the use of such complicated mathematical calculations that make the operation longer and more complicated is not necessary. Our study showed no cases of insufficient lengthening or over-lengthening.

According to one report, deformity recurrence was observed in 4% of patients from the percutaneous group and in 21.4% of patients from the Z-plasty group [2]. Borowski et al. observed deformity recurrence in 20.46% of patients who had undergone percutaneous Achilles tendon lengthening [3]. Zhang et al. reported a recurrence rate of 5.7% [11], which is identical to that observed in our patients from the Z-plasty group, with no cases of deformity recurrence observed in the percutaneous lengthening group.

In our study, the complication rates were 4% and 11.4% in patients from the percutaneous lengthening group and from the Z-plasty group, respectively, which is a slightly better result than those reported in the literature [1,2,3,10,11,13,15]. Some reports suggested Z-plasty to be more likely to result in blood vessel damage and cause Achilles tendon rupture in comparison with the percutaneous lengthening technique [11]. In our study, there were no cases of blood vessel damage or Achilles tendon rupture in either of the evaluated groups. Moreover, we observed no cases of calcaneal deformity, swelling, infection, delayed wound healing, soft tissue necrosis, adhesion, scarring, blood vessel or nerve damage, or joint instability, which indicates good treatment outcomes.

In the group of 19 patients undergoing percutaneous Achilles tendon lengthening assessed by Kha et al., 26.3% of patients continued to periodically walk on their toes after the procedure, and 11% of patients had constant gait abnormalities [1]. Berg et al. observed a normal gait in all the patients who had undergone percutaneous Achilles tendon lengthening [13]. In our study, 100% of patients had gait abnormalities prior to treatment. At long-term post-treatment follow-up, abnormal gait was observed in two patients (5.7%) from the Z-plasty group and in none of the patients from the percutaneous lengthening group, which indicates good treatment outcomes. Our study results are comparable with those reported in the literature [1,13].

Lin et al. noted a mean post-treatment AOFAS score of 96.08 points in the group treated with modified percutaneous lengthening and a mean score of 85.4 in the group treated with Z-plasty [2]. A study by Hao et al. demonstrated a change in the AOFAS score from 60.5 prior to treatment to 90.8 after treatment [10]. Another study evaluating patients undergoing percutaneous Achilles tendon lengthening revealed an increase in the AOFAS score from 64.97 prior to the procedure to 90.06 at follow-up [11]. Kim et al. reported an improvement in AOFAS scores from 56.1 before to 81.8 after treatment [12].

The patients from both of our study groups achieved a significant improvement in terms of post-treatment AOFAS scores, which indicates good outcomes for both surgical techniques. Moreover, the two study groups showed no differences in post-treatment AOFAS scores. These results were comparable with those reported by other authors who used the AOFAS scale to assess Achilles tendon lengthening outcomes [2,10,11,12].

There are no available reports on such important issues as the use of painkillers prior to and after Achilles tendon lengthening. Leyko et al. assessed the use of painkillers in patients following hemiepiphysiodesis [17]. Those authors reported a preoperative proportion of analgesic-using patients of 14%, with no patients using such medications after treatment [17]. Taking painkillers before surgery was reported in 11.4% of patients from the Z-plasty group and in 4% of patients from the percutaneous lengthening group; the difference was not statistically significant. Follow-up assessments showed no patients from either group taking painkillers, which may be an indicator of good functional outcomes.

Out of the patients undergoing percutaneous Achilles tendon lengthening, Kha et al. reported the use of normal footwear by 95% of patients after treatment [1]. In our study, all the patients from both groups used normal footwear after treatment, which suggests good functional outcomes of the evaluated surgical techniques.

Another not fully explored aspect associated with Achilles tendon lengthening is the precise extent of post-treatment improvement in ankle dorsiflexion. The authors of some previous studies reported that the percutaneous lengthening technique and Achilles tendon Z-plasty improve the range of ankle dorsiflexion; however, those authors did not assess the baseline and/or the postoperative range of ankle dorsiflexion [2,5,11]. In our study, the percutaneous lengthening group achieved postoperative ankle dorsiflexion improvement by 30°, assessed at a median follow-up of 33 months after the procedure. In the Z-plasty group, the improvement in ankle dorsiflexion, measured after a median of 26 months after the procedure, was 32.5°. There was no significant difference in ankle dorsiflexion improvement between the groups. Kha et al. reported an increased range of ankle dorsiflexion by a mean of 28° after percutaneous Achilles tendon lengthening [1]. Blumel observed improved ankle dorsiflexion by a mean of 21° after percutaneous lengthening [9]. In a study by Hao et al. the mean improvement in ankle dorsiflexion was 24° after endoscopic Achilles tendon lengthening [10]. Another study assessing various techniques of Achilles tendon lengthening demonstrated post-treatment ankle dorsiflexion improvement by a mean of 27° [12]. Berg observed a mean improvement in ankle dorsiflexion by 30° following percutaneous lengthening [13]. The patients assessed by Hemo et al. showed an improvement in ankle dorsiflexion by a mean of 19° at postoperative follow-up [15]. The extent of ankle dorsiflexion improvement achieved in both groups in our study was similar to or slightly better than those reported by other authors [1,9,10,12,13,15].

In our study, the group of patients who underwent Achilles tendon lengthening with the conventional percutaneous technique showed a median ankle dorsiflexion improvement of 30°. The extent of ankle dorsiflexion improvement achieved in the percutaneous lengthening group in our study does not differ from that reported in studies assessing a modified percutaneous lengthening technique (ankle dorsiflexion improvement by 27°) [12]. Our study results promote the use of the conventional percutaneous lengthening technique, which is characterized by a shorter procedure duration, easier surgical technique, smaller surgical incisions, lower risk of complications, and a comparable improvement in ankle dorsiflexion. Moreover, while comparing our treatment outcomes in the group undergoing conventional percutaneous lengthening of the Achilles tendon with those reported by other authors, who assessed modified percutaneous techniques [2,11,12], we observed lower total complication rates, fewer cases of deformity recurrence, no cases of toe walking after surgery, a shorter duration of cast immobilization, and comparable AOFAS scores, which also advocates for the use of the conventional percutaneous lengthening technique.

One of the limitations of our study was its retrospective nature, which was a result of our aim to quickly present the outcomes of Achilles tendon lengthening via a percutaneous technique or Z-plasty in a relatively large group of patients. Other authors who assessed Achilles tendon lengthening also conducted retrospective studies [1,2,3,4,5,9,10,11,15]. Another limitation of our study was the small size of our study population; however, papers by other authors were based on similar or even smaller groups of patients undergoing Achilles tendon lengthening [1,2,3,4,6,9,10,11,13,14,15]. Given the retrospective nature of our study and the small size of the study groups, the results should be interpreted with caution. The retrospective design and the fact that the surgical technique was selected according to surgeon preference introduce a risk of selection bias. Since each surgical technique was exclusively performed by a different surgeon, the effects of surgical technique and operating surgeon cannot be separated; this is an important limitation. A potential confounding factor was the inclusion of patients undergoing bilateral tendon lengthening in the study. However, other publications have also evaluated subsets of patients undergoing bilateral tendon lengthening without division into groups with unilateral and bilateral lengthening [2,3,4,9,10,12,13]. In our cohort, only 5 out of 60 patients underwent bilateral tendon lengthening (three in the Z-plasty group and two in the percutaneous lengthening group). Patients undergoing bilateral tendon lengthening were included in order to increase the total number of patients evaluated. Baseline differences between the two treatment groups may have influenced the observed outcomes. Since the percutaneous group had significantly better preoperative ankle dorsiflexion than the Z-plasty group, and no baseline-adjusted analysis was performed, postoperative comparisons should be interpreted with caution. The between-group findings are exploratory and may be influenced by baseline and surgeon-related confounding. The limitations of our study also include the lack of randomization and the absence of gait analysis.

Since the etiology of Achilles tendon abnormality to be corrected by lengthening may affect the results of the procedure [9,12,15], in our study, we assessed only patients with idiopathic Achilles tendon contracture; this is one of the strengths of our study. Another strength of our study is the study population within a specific age range (5–17 years), which included only pediatric patients. Other authors assessed populations with much greater age differences, often indiscriminately analyzing both children and adults. Example patient age ranges in other studies include 5–43 years [2], 2–47 years [5], 16–48 years [10], 14–36 years [11], and 4–28 years [12], which may potentially confound outcome assessment. In the future, we are planning to conduct prospective studies in a larger patient population, studies on a group of adult patients, gait analysis, and with a longer follow-up period. Given that blockchain technology is useful for managing patient registry and diagnostic imaging data, including in orthopedics [19], we plan to conduct research utilizing blockchain technology in the future.

Both of the groups assessed in our study had similar periods of immobilization, overall complication rates, deformity recurrence rates, AOFAS scores, levels of satisfaction with treatment, proportion of patients who would choose the same treatment method again, duration of hospital stay, improvement in ankle dorsiflexion, use of painkillers, and the absence of Achilles tendon rupture and toe walking. Our study results suggest that both evaluated techniques are effective methods for lengthening the Achilles tendon in children with idiopathic contracture.

## 5. Conclusions

The period of cast immobilization of six weeks helped achieve good outcomes and caused few complications in children from both study groups.

The conventional percutaneous approach to Achilles tendon lengthening in children helps achieve substantial correction of ankle dorsiflexion with a low risk of complications.

Our study results suggest that both evaluated techniques are effective methods for lengthening the Achilles tendon in children with idiopathic contracture.

Due to the risk of selection bias, the results should be interpreted with caution.

## Figures and Tables

**Figure 3:**
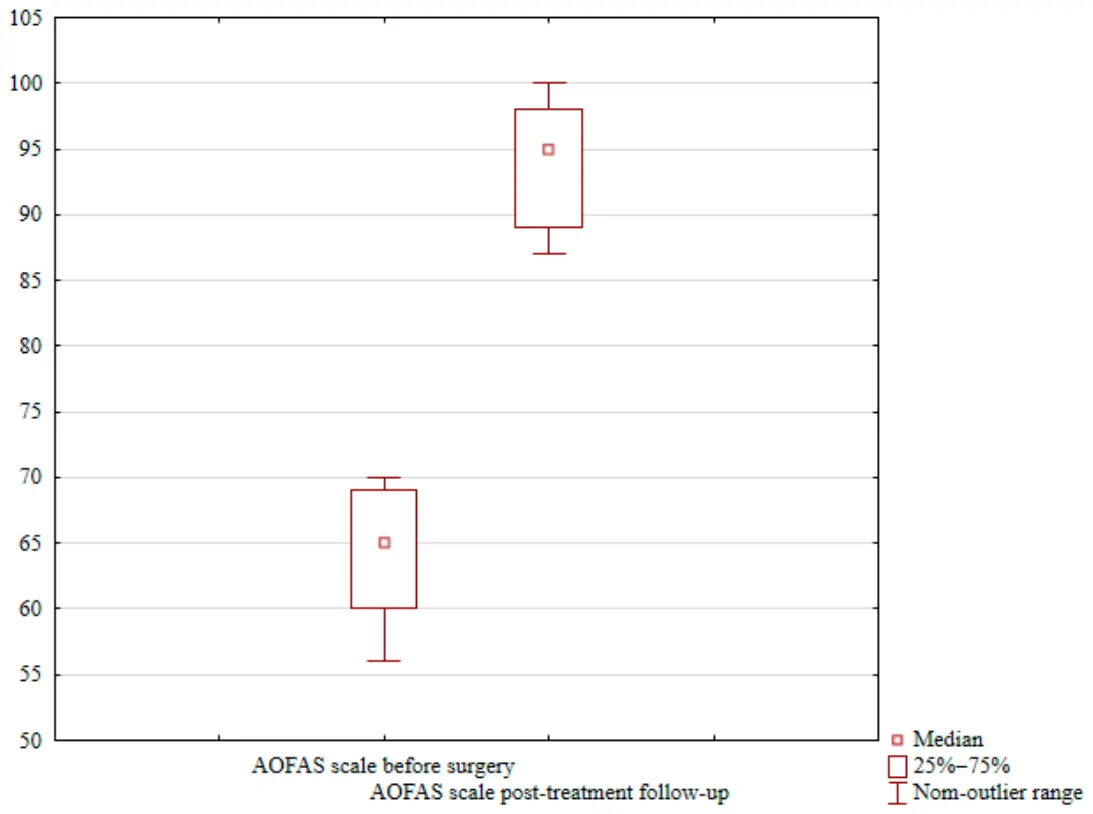
AOFAS scale scores before surgery and in post-treatment follow-up in the percutaneous lengthening group.

**Table 1:** Detailed data of patients from the Z-lengthening group and percutaneous group.

Analyzed Variable	Group	Q1	Median	Q3	Z	*p*	Effect Size r	Approx. Cohen’s d
Age [years]	Z-lengthening	9	11	13	2.721	0.070	0.35	0.75
	Percutaneous	9	10	12				
Follow-up [months]	Z-lengthening	16	26	43	−0.952	0.341	0.12	0.24
	Percutaneous	17	33	45				
Hospitalization period [days]	Z-lengthening	3	3	3	0.376	0.707	0.05	0.10
	Percutaneous	3	3	3				
Cast maintenance period [weeks]	Z-lengthening	6	6	6	0.437	0.662	0.06	0.11
	Percutaneous	6	6	6				
Time to return to normal activity [weeks]	Z-lengthening	6	6	7	2.258	0.024	0.32	0.67
	Percutaneous	6	6	6				
AOFAS before surgery	Z-lengthening	59	66	69	−0.255	0.799	0.03	0.06
	Percutaneous	60	65	69				
AOFAS during the post-treatment follow-up	Z-lengthening	88	95	98	−0.712	0.476	0.09	0.18
	Percutaneous	89	95	98				
Ankle dorsiflexion before surgery [degree]	Z-lengthening	−15	−7.5	0	−2.321	0.020	0.40	0.87
	Percutaneous	−5	0	5				
Ankle dorsiflexion post-treatment follow-up [degree]	Z-lengthening	20	25	30	−1.985	0.047	0.35	0.74
	Percutaneous	25	30	30				
				Z-lengthening group	Percutaneous group	χ^2^	*p*	Effect size
Taking painkillers before surgery	% of observations			11.4	4.0	1.054	0.305	φ = 0.133
Taking painkillers during the post-treatment follow-up				0.0	0.0	0.000	1.000	φ = 0.000
The same treatment method again				94.3	100.0	1.478	0.224	φ = 0.157
Complications				11.4	4.0	1.054	0.305	φ = 0.133
Patient very satisfied with the treatment				68.6	88.0	3.281	0.194	V = 0.234
Patient satisfied with the treatment				28.6	12.0	3.281	0.194	V = 0.234
Caregiver very satisfied with the treatment				77.1	92.0	2.501	0.286	V = 0.204
Caregiver satisfied with the treatment				20.0	8.0	2.501	0.286	V = 0.204
Equinus recurrence				5.7	0.0	1.478	0.224	φ = 0.157
Achilles tendon rupture				0.0	0.0	0.000	1.000	φ = 0.000
Calcaneal gait				0.0	0.0	0.000	1.000	φ = 0.000
Abnormal gait before surgery				100.0	100.0	0.000	1.000	φ = 0.000
Abnormal gait during the post-treatment follow-up				5.7	0.0	1.478	0.224	φ = 0.157

Legend: Q1, Q3—1st and 3rd quartiles; Z—standardized value of the Mann–Whitney U test; χ^2^—chi-square test statistic; *p*—significance level; r—effect size for the Mann–Whitney U test (r = ∣Z∣/N); d—approximate conversion from r to Cohen’s d (d = 2r/1 − r2), interpreted with caution for non-parametric data; φ—Phi coefficient for 2 × 2 contingency tables; V—Cramér’s V coefficient for contingency tables larger than 2 × 2. Effect size interpretation: small (0.10), medium (0.30), large (0.50).

**Table 2:** Results before surgery and at last follow-up.

Analyzed Variable	Value	Before Surgery	Last Follow-Up	Z	*p*	Effect Size r
**Z-lengthening group**						
AOFAS	Q1	59	88			
	Median	66	95	5.159	<0.001	0.87
	Q3	69	98			
Ankle dorsiflexion [degrees]	Q1	−15	20			
	Median	−7.5	25	4.860	<0.001	0.87
	Q3	0	30			
						Effect size, g
Use of analgesics [%]		11.4%	0.0%	McNemar exact	1.000	0.114
Abnormal gait [%]		100.0%	5.7%	McNemar exact	<0.001	0.943
**Percutaneous group**						
AOFAS	Q1	60	89			Effect size r
	Median	65	95	4.372	<0.001	0.87
	Q3	69	98			
Ankle dorsiflexion [degrees]	Q1	−5	25			
	Median	0	30	4.107	<0.001	0.88
	Q3	5	30			
						Effect size, g
Use of analgesics [%]		4.0%	0.0%	McNemar exact	1.000	0.04
Abnormal gait [%]		100.0%	0.0%	McNemar exact	<0.001	1

Q1—lower quartile; Q3—upper quartile; Z—Wilcoxon signed-rank test statistic; *p*—significance level; r—effect size for the Wilcoxon signed-rank test calculated as r = Z/√N. Categorical paired variables were analyzed using the exact McNemar test. Cohen’s g was used as the effect size measure for paired binary outcomes and calculated as g = |b − c|/N, where b and c denote the discordant pairs.

## Data Availability

The data presented in this study are available on request from the corresponding author.
